# Postoperative gastric cancer accompanied by large-cell neuroendocrine carcinoma: A case report

**DOI:** 10.1097/MD.0000000000044367

**Published:** 2025-10-10

**Authors:** Zhiqin Chen, Jiang Liu, Jin Liu, Yinhang Wu, Jian Liu

**Affiliations:** a Huzhou Central Hospital, Affiliated Central Hospital Huzhou University, Huzhou City, Zhejiang Province, People’s Republic of China; b Zhejiang-France United Laboratory of Integrated Traditional Chinese and Modern Medicine in Colorectal Cancer, Huzhou City, Zhejiang Province, People’s Republic of China; c Department of Gastroenterology, Huzhou Central Hospital, Fifth School of Clinical Medicine of Zhejiang Chinese Medical University, Huzhou City, Zhejiang Province, People’s Republic of China.

**Keywords:** gastric cancer liver metastasis, large-cell neuroendocrine carcinoma, pathology, postoperative gastric cancer, treatment

## Abstract

**Rationale:**

Large cell neuroendocrine carcinoma (LCNEC) is a type of neuroendocrine carcinoma that is a rare malignant tumor that often coexists with adenocarcinoma in the same tissue. Adenocarcinoma cells may transform into LCNEC cells. The patient was found to have a liver mass 3 years after gastric adenocarcinoma surgery. Due to the absence of neuroendocrine cells in the liver, the patient’s liver lesions may undergo neuroendocrine transformation after adenocarcinoma metastasis.

**Patient concerns:**

The patient was found to have a liver mass 3 years after gastric adenocarcinoma surgery, and the preliminary clinical diagnosis was gastric cancer with liver metastasis. The patient has no discomfort symptoms, and the relevant physical examination shows no abnormalities.

**Diagnoses:**

Based on the patient’s medical history, the preliminary clinical diagnosis is gastric cancer with liver metastasis (not excluding the possibility of primary LCNEC of the liver).

**Interventions:**

The patient received surgical treatment and comprehensive treatment, including EP regimen chemotherapy and promotion of white blood cell growth. The patient received regular postoperative chemotherapy.

**Outcomes:**

The patient is currently undergoing regular outpatient follow-up. The patient was last followed up on September 18, 2024.

**Lessons:**

This patient is a rare case of large liver cell neuroendocrine carcinoma after radical gastrectomy for gastric cancer, as the liver does not have neuroendocrine cells. The origin of liver neuroendocrine carcinoma is still unclear. Therefore, whether the patient was diagnosed with secondary liver metastasis of gastric cancer or concurrent liver in situ LCNEC is a difficult question. We hope to assist in solving relevant clinical problems through the special patient’s situation.

## 1. Introduction

Neuroendocrine carcinoma (NEC) is a rare and invasive tumor that typically combines multiple histological features, known as mixed neuroendocrine non-neuroendocrine tumor (MiNEN).^[1]^ Among them, adenocarcinoma is closely related to neuroendocrine carcinoma, as neuroendocrine carcinoma often coexists with adenocarcinoma tissue, and some adenocarcinoma tissues may undergo neuroendocrine transformation.^[2]^

Neuroendocrine transformation refers to the phenotypic transformation of non-neuroendocrine tumors into neuroendocrine tumors, which often occurs in adenocarcinoma. Both lung and prostate adenocarcinoma can undergo a neuroendocrine transformation.^[3–5]^ This patient is a rare case of postoperative gastric cancer complicated with hepatic neuroendocrine carcinoma, as the liver does not have neuroendocrine cells. Hence, the origin of hepatic neuroendocrine carcinoma is not clear. This case introduces the patient’s clinical and pathological characteristics, diagnosis, and treatment process, and discusses and analyzes the patient’s unique situation.

## 2. Patient information

**Figure 1.**
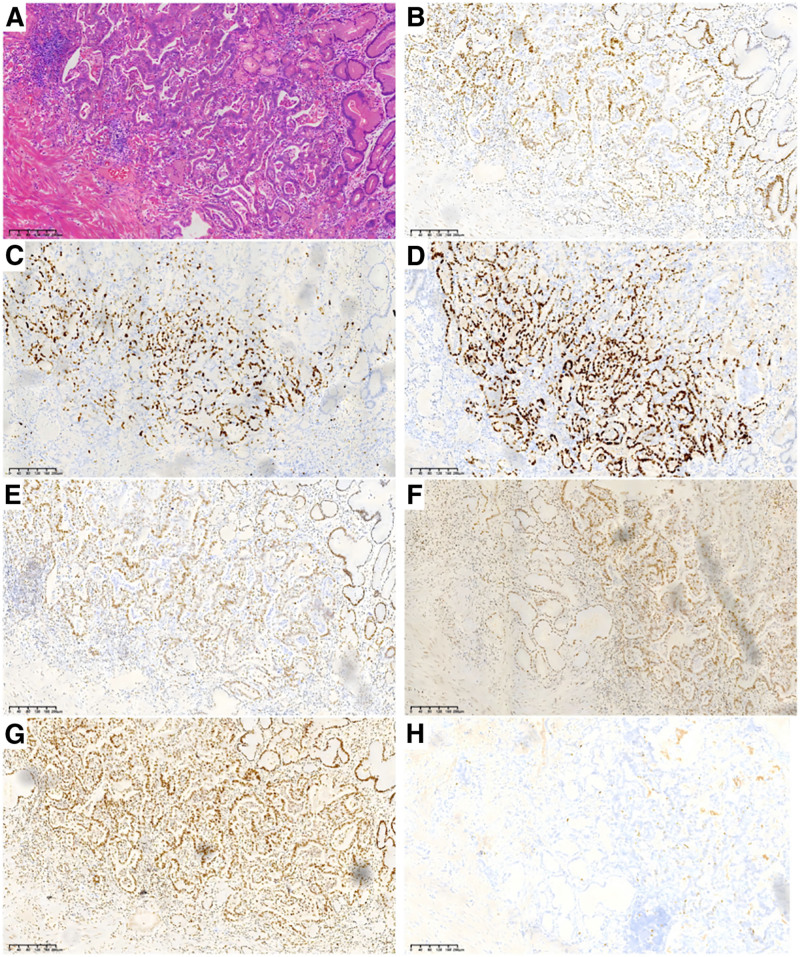
shows the HE-stained pathological images and immunohistochemical results of the patient after radical gastrectomy for gastric cancer. (A) show of HE-stained pathological images. Manifestations of moderately to poorly differentiated adenocarcinoma. Immunohistochemical results: (B) shows MSH6 (+), (C) shows Ki-67 (+, about 20%), (D) shows P53 (3+), (E) shows MLH1 (+), (F) shows PMS2 (+), (G) shows MSH2 (+), (H) shows Topo II (1+). (The scanning magnification of the above image is: 10X).

The patient is a 73-year-old retired Han male who was hospitalized 5 years ago due to a reported “choking sensation after eating for over 6 months.” During hospitalization, an abdominal CT scan showed “occupying space at the bottom of the stomach and on the lesser curvature side, with enlarged lymph nodes in the hepatogastric space.” Gastroscopy pathology revealed moderately to poorly differentiated adenocarcinoma (in the gastric corner) and moderately to poorly differentiated adenocarcinoma (in the gastric cardia). The patient was diagnosed with advanced gastric cancer. The FLOT regimen is often used as a perioperative treatment for gastric cancer. The study by Gökhan Uçar et al^[6]^ showed that 7.8% of patients achieved complete pathological remission (n = 103) under the FLOT regimen, and their average survival time was 30.9 months. The patient received 3 rounds of FLOT regimen chemotherapy on May 21, June 4, and June 18, 2020, and the process went smoothly. After excluding contraindications, a laparoscopic radical gastrectomy (total gastrectomy + Roux-en-Y esophagojejunal anastomosis) was performed on July 9, 2020. The surgery took 4 hours, with intraoperative bleeding of 50ml. Postoperatively, hypoalbuminemia occurred, which improved after albumin supplementation. The patient was discharged 14 days after surgery. Postoperative pathological results showed ulcerative adenocarcinoma of the gastric cardia (mass size 4.5 × 4 cm) (Fig. 1). The patient received the FLOT regimen chemotherapy 4 times in the oncology department on September 17, October 10, October 27, and December 4, 2020, specifically: docetaxel 70 mg dL + oxaliplatin 0.12 g dL + calcium folinate 0.3 g dL + fluorouracil 3.75g ci v46h” chemotherapy. The process went smoothly. Grade III bone marrow suppression (N minimum 0.7 × 10^9^/L) occurred after chemotherapy. The patient’s bone marrow suppression improved after receiving treatment to promote white blood cell growth. Discharge and instruct the outpatient department to conduct regular follow-up examinations.

The patient has a history of smoking for over 20 years and was allergic to penicillin. 14 years ago, he underwent inguinal hernia surgery. His older brother has a history of gastric cancer, but no other medical history is significant.

The patient underwent a full abdominal CT scan on November 10, 2023, which showed a new lesion in the left lobe of the liver (Supplementary Material 1, Supplemental Digital Content, https://links.lww.com/MD/Q19). The patient was therefore readmitted.

## 3. Clinical findings

After admission, the patient completed upper abdominal MR (Supplementary Material 2, Supplemental Digital Content, https://links.lww.com/MD/Q19) and PET-CT(Supplementary Material 3, Supplemental Digital Content, https://links.lww.com/MD/Q19), and the examination results showed the presence of a left lobe liver mass. No signs of metastasis were found in other organs. The patient showed no discomfort symptoms before or after these examinations, and there were no positive results in the relevant physical examinations. After completing relevant examinations and excluding contraindications, the patient underwent a “B-ultrasound-guided liver biopsy” on November 27, 2023. The biopsy pathology showed “metastatic or invasive cancer in the left liver biopsy specimen, and immunohistochemical testing is recommended.” Cancer cells were found (positive) in the left liver biopsy cytology smear, and metastasis was not ruled out.”

## 4. Timeline

**Figure 2.**
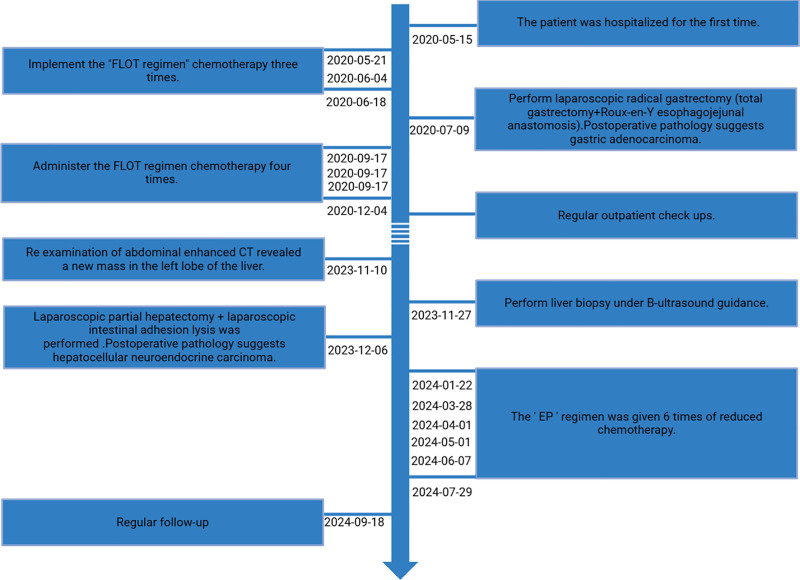
shows the patient’s medical timeline from diagnosis to the latest treatment of the patient, as of the time reported in the literature.

Figure 2 illustrates the patient’s medical timeline from diagnosis to the latest treatment reported in the literature, as of the time indicated.

## 5. Diagnostic assessment

Based on the patient’s pathological results, imaging examinations, and medical history, it is considered that the patient has gastric cancer liver metastasis accompanied by neuroendocrine transformation (pT3N2M1, stage IV). However, the patient cannot completely rule out the possibility of a second primary tumor.

The patient has a rare postoperative gastric cancer complicated with hepatic neuroendocrine carcinoma, as the liver lacks neuroendocrine cells, and there have been previous reports of neuroendocrine transformation. So the origin of the liver lesion in this patient is not completely clear.

## 6. Therapeutic intervention

**Figure 3.**
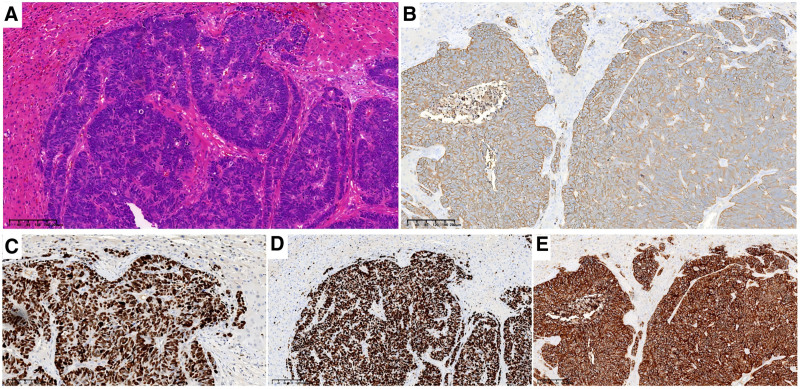
shows the HE-stained pathological images and immunohistochemical results of the patient’s left liver wedge resection specimen. (A) show of HE-stained pathological images. Lowly differentiated cancer, with cancer cells appearing as large cells. Immunohistochemical results: (B) displays SYN (3+), (C) displays P53(2+), (D) displays Ki-67(+about 80%), (E) displays CD56 (3+) (The scanning magnification of the above image is: 10X).

The patient was transferred to the Hepatobiliary Pancreatic Surgery Department and underwent“ laparoscopic partial hepatectomy + laparoscopic intestinal adhesiolysis “under general anesthesia on December 6, 2023. The patient underwent non-anatomical resection of the liver lesion. The surgery took 2 hours and 50 minutes, with intraoperative bleeding of approximately 50 mL. Postoperatively, hypoalbuminemia occurred, which improved after albumin supplementation. The patient was discharged 12 days after surgery. Postoperative pathology revealed low-differentiation cancer with LCNEC changes (maximum lesion diameter, 2.5 cm) (Fig. 3).

According to the CACA Neuroendocrine Tumor 2022 Guidelines, for neuroendocrine cancers with unclear primary lesions and poor differentiation, postoperative chemotherapy is recommended and the EP regimen can be adopted. EP regimen has shown good efficacy in treating small-cell neuroendocrine carcinoma, with a median survival of 289 days.^[7]^ Recent studies have shown that among patients with pure LCNEC, those who receive chemotherapy regimens for small cell neuroendocrine carcinoma have a better prognosis, with a median survival of 19.5 months.^[8]^

After obtaining informed consent from the patient and their family, the doctor determined the implementation of the postoperative EP plan. The patient started the first cycle of “etoposide 0.12 d1-3 + cisplatin 30mg d1-3, q3w” reduced chemotherapy (standard dose 80%) on January 22, 2024. After chemotherapy, there were grade I gastrointestinal reactions and obvious hiccups, and the patient felt intolerant and postponed chemotherapy. After the first cycle of treatment, grade III bone marrow suppression (with a minimum N value of 0.7 × 10^9^/L) occurred, which improved after treatment with treatment that promotes white blood cell growth. On March 8, 2024, April 1, 2024, and May 1, 2024, the original dosage of the original regimen was administered for the second to fourth cycles of chemotherapy. The process went smoothly, with no recurrence of hiccups or obvious discomfort such as nausea and vomiting. After chemotherapy, Treatment has been administered to promote white blood cell growth. Starting from June 7, 2024, the “EP” regimen of reduced chemotherapy (standard dose 80%) will be administered, supplemented with antiemetic, gastric protection, and fluid replacement treatments. Two days after chemotherapy, blood and kidney function tests showed an increase in creatinine compared to before. Chemotherapy was canceled on the third day, and after fluid replacement support treatment, blood and kidney function improved. On June 10, 2024, the patient was given prophylactic treatment that promotes white blood cell growth with “Jinyouli 6mg.” The patient’s general condition was normal without obvious nausea, vomiting, or discomfort. The patient was discharged on June 11, 2024. The patient was hospitalized on July 29, 2024, for the sixth round of chemotherapy. Following the original chemotherapy regimen, the patient did not experience any significant discomfort after chemotherapy. Discharged on July 31, 2024, with regular outpatient follow-up.

## 7. Follow-up and outcomes

By fully communicating and exchanging ideas with patients and their families, a consensus can be reached on the treatment plan, thereby achieving high compliance. Evaluate the patient’s tolerance to treatment based on their reactions during the treatment process and related examinations, such as blood routine and liver and kidney function test results. After comprehensive treatment, the patient’s overall condition is good. There were no adverse or unexpected events during the patient’s treatment process. Currently, there has been no tumor recurrence during the follow-up period, and the treatment effect is satisfactory.

The patient is currently undergoing regular outpatient follow-up. The patient was last followed up on September 18, 2024.

## 8. Discussion

This case reports a rare case of gastric adenocarcinoma complicated with hepatic large-cell neuroendocrine carcinoma. Due to the absence of parenchymal neuroendocrine cells in the liver, identifying the origin of large hepatic cell neuroendocrine carcinoma has become a challenging task. We will discuss this case, and based on the patient’s special circumstances, changes in the patient’s condition, progression, and treatment may provide a reference for the diagnosis and treatment of similar patients. Limitations: The clinical diagnosis of this case suggests gastric adenocarcinoma complicated with liver large-cell neuroendocrine carcinoma metastasis, but based on the current known situation, the origin of the liver lesion in this patient is still unclear.

Large-cell neuroendocrine carcinoma is a highly invasive neuroendocrine carcinoma, commonly found in the lungs, followed by the pancreas, gastrointestinal tract, etc, and extremely rare in other areas.^[9,10]^ Primary neuroendocrine carcinoma of the liver is rare, accounting for only 0.3% of all neuroendocrine tumors.^[11]^ Primary hepatic LCNEC is a type of primary hepatic neuroendocrine carcinoma, which is rare.^[12,13]^ Due to the limited research on LCNEC of the liver, its origin^[14]^ and treatment are still unclear.^[15,16]^

LCNEC of the liver is difficult to distinguish from other liver tumors through ultrasound, CT, MRI, and other examinations. Most neuroendocrine cancers are nonfunctional, and only a small number of patients may exhibit endocrine disorders such as hypercalcemia.^[17]^ Therefore, it is difficult to diagnose and differentiate based on clinical manifestations. Most patients are diagnosed through pathological results.^[18]^ Through routine pathological observation, changes in large, small, and mixed tissues can be distinguished by the microscopic appearance of cell morphology lesions. Combined with pathological HE staining under the microscope, immunohistochemistry of relevant neuroendocrine markers such as CGA, SYN, etc. can assist in diagnosis. SYN has a sensitivity of 85% −100% to neuroendocrine cells. However, its specificity is low and it can also be expressed in some adenocarcinoma or squamous cell carcinoma cells.^[19–21]^ CGA has lower sensitivity than SYN, but higher specificity than SYN.^[21,22]^

The main distinguishing factor between primary hepatocellular neuroendocrine carcinoma and metastatic carcinoma is the presence of the primary lesion. When 2 large-cell neuroendocrine carcinoma lesions are discovered simultaneously, due to the rarity of primary large-cell neuroendocrine carcinoma of the liver and the absence of neuroendocrine cells in the liver, considering the high possibility of metastatic tumors, genetic testing of lesion tissue may be helpful for diagnosis^.[23,24]^

The high incidence rate of hepatocellular carcinoma provides clinical data support for its research progress in diagnosis and treatment.^[25]^ In contrast, the rarity of LCNEC cases has led to a lack of clarity regarding treatment options and outcomes.Currently, it is widely believed that surgical treatment is the main treatment method for focal lesions.^[16]^ There are also reports that the combination therapy of “oxaliplatin + gemcitabine + carragelizumab + apatinib” is effective in treating the LCNEC of the liver.^[15]^ Hiroko Hasegawa and colleagues have also reported cases of primary large-cell neuroendocrine carcinoma with tumor reduction and prolonged survival after chemotherapy with etoposide and cisplatin.^[26]^ Due to the rarity of related cases, research on liver large-cell neuroendocrine carcinoma is not yet in-depth, and immunotherapy and molecular targeted therapy have not been applied in the treatment of liver large-cell neuroendocrine carcinoma. However, due to the rarity of clinical cases, its treatment lacks precise and feasible clinical trials and evidence-based medical support.

The liver does not have neuroendocrine cells, but clinical cases have shown that neuroendocrine tumors, including LCNEC, can originate in the liver. The origin of primary LCNEC in the liver is not yet clear. There are currently 3 main hypotheses: neuroendocrine-like cells in intrahepatic bile duct epithelial cells, which have been previously found in rat liver,^[27]^ In addition, neuroendocrine cells can be detected in the site of chronic inflammation-induced intestinal metaplasia caused by gallstones and congenital abnormalities.^[28–30]^ However, not all previous reports on neuroendocrine tumors have included chronic inflammation and intestinal epithelium due to biliary obstruction.^[31]^ Tumors originate from displaced pancreatic or adrenal cells. The existence of mixed non-neuroendocrine neuroendocrine liver tumors suggests the possibility of differentiation from non-neuroendocrine or stem cell-like precursor cells into neuroendocrine cells,^[11,13]^ Moreover, patients with hepatic neuroendocrine carcinoma accompanied by other types of cancer tissue may have more cases compared to those with simple hepatic neuroendocrine carcinoma.^[32–35]^

This case has its particularity. The patient has a history of gastric cancer, and there may be doubts about the source of LCNEC in the patient’s liver. The patient’s liver LCNEC may be the second primary tumor after gastric cancer, and there is also a possibility of gastric cancer liver metastasis. The clinical diagnosis of the second primary cancer (SPC) mainly includes 3 points: Each tumor must be malignant; There is a certain distance between each tumor; It must be ruled out that 1 tumor is a metastatic tumor of another tumor. Subsequent supplements were made to the second and third points: recurrent and indicator tumors have similar histological features, and both have a normal epithelial gap of at least 2 cm and/or occur at least 3 years after the indicator tumor. Recurrent tumors from different histological sources are also considered SPC.^[36]^ The incidence of gastric adenocarcinoma complicated with a second primary tumor is very low. Liyan Jin et al^[37]^ study (including 4041 patients) showed that the probability of developing SPC is only 4.3%. Only 2.5% of patients with concurrent SPC have liver tumors as their second tumor. The second primary tumor in the liver is often hepatocellular carcinoma.^[38]^ The second primary tumor is rarely liver large-cell neuroendocrine carcinoma, and there have been no reports in the relevant literature so far. The liver is a common site of gastric adenocarcinoma metastasis. Clinically, liver lesions are considered to occur within <3 years after gastric adenocarcinoma surgery, and there are no neuroendocrine cells in the liver, with the possibility of neuroendocrine transformation. Considering the likelihood of gastric adenocarcinoma liver metastasis with neuroendocrine differentiation of cancer cells in this patient. Although the pathological type of metastatic lesions is often consistent with that of the primary lesion, pathological changes are possible. LCNEC can often coexist with adenocarcinoma and form mixed gonadal neuroendocrine carcinoma in the same organ.^[28,39–41]^ Gastric cancer itself has neuroendocrine cells, and there is a case of gastric cancer with neuroendocrine differentiation (NEDGC) in the gastric cancer types. Kazuto Yamazaki^[42]^ reported a case of gastric cancer sarcoma with neuroendocrine cell differentiation. Through the study of immunohistochemistry and ultrastructure of the patient, it was suggested that the patient may have developed from conventional tubular adenocarcinoma. Andrew J Syder et al^[43]^ described a transgenic mouse model of meta-analysis of gastric cancer initiated by expressing Simian virus 40 large tumor antigen (SV40 TAg), under the control of regulatory elements from the mouse Atp4b gene, in the progenitors of acid-producing parietal cells. Through the study of this model, Andrew J Syder discovered that certain non-neuroendocrine cells could undergo neuroendocrine regulation through the regulation of specific genes. In addition, there are also cases of gastric adenocarcinoma with lymph node LCNEC metastasis, and no other tumor lesions have been reported.^[44]^ The neuroendocrine carcinoma of this lymph node may have metastasized from a gastric adenocarcinoma lesion. Lung adenocarcinoma and prostate adenocarcinoma can undergo a neuroendocrine transformation, transforming some adenocarcinoma tissues into small-cell neuroendocrine carcinoma or large-cell neuroendocrine carcinoma. The mechanism of large or small-cell neuroendocrine transformation in lung adenocarcinoma may be consistent, mostly occurring after Immunotherapy.^[45]^ Most SCLC-transformed patients have RB and TP53 inactivation mutations.^[46,47]^ In addition, studies have found that DPYSL5,^[4]^ Exportin1,^[48]^ EMHT2,^[49]^ PI3K/AKT pathway,^[50]^ and others play important roles in neuroendocrine transformation in lung adenocarcinoma and prostate adenocarcinoma. Next-generation sequencing of mixed hepatocellular NEC and small cell NEC in liver tumors revealed common molecular/genetic alterations in hepatocellular carcinoma (HCC). Indicating that a portion of hepatocellular carcinoma may undergo neuroendocrine transformation.^[24,51]^ These indicate that gastric adenocarcinoma may undergo neuroendocrine transformation similar to lung adenocarcinoma and prostate cancer, and its mechanism of occurrence may be similar.In addition, the tumor microenvironment can cause changes in the immunosuppressive phenotype of gastric cancer,^[52]^ and perhaps changes in the tumor microenvironment play a role in the transformation of gastric adenocarcinoma.

The patient underwent postoperative “FLOT regimen” chemotherapy. Will this chemotherapy affect the neuroendocrine differentiation of gastric cancer and lead to the occurrence of LCNEC? This may also be a question worth considering. The “FLOT regimen” chemotherapy is a commonly used chemotherapy regimen for gastric cancer, and it is currently believed that implementing the “FLOT regimen” chemotherapy during the perioperative period of gastric cancer is beneficial for patients.^[6,53,54]^ However, there is currently little research on whether the FLOT regimen of chemotherapy can lead to neuroendocrine differentiation in gastric cancer, and it is impossible to prove the role of chemotherapy in this process.

We believe that for the study of the origin of liver LCNEC, we may refer to Andrew J Snyder^[42]^ research to investigate the neuroendocrine differentiation mechanism of the liver from the perspective of gene and molecular regulation. Combined with previous studies on the existence of neuroendocrine-like cells in obstructive biliary diseases, we can use this as a starting point to explore the origin of liver neuroendocrine tumors.

The patient is currently being followed up with an overall survival of over 4 years and a progression-free survival of over 8 months. Long-term management is necessary for such patients. This may include managing diet and lifestyle habits, as well as closely monitoring changes in the patient’s condition during radiotherapy and chemotherapy.

This case reports a rare case of gastric adenocarcinoma complicated with hepatic large-cell neuroendocrine carcinoma. Due to the absence of parenchymal neuroendocrine cells in the liver, identifying the origin of large hepatic cell neuroendocrine carcinoma has become challenging. We will discuss this case, and based on the patient’s special circumstances, changes in the patient’s condition, progression, and treatment may provide a reference for the diagnosis and treatment of similar patients. Limitations: The clinical diagnosis of this case suggests gastric adenocarcinoma complicated with liver large-cell neuroendocrine carcinoma metastasis, but based on the current known situation, the origin of the liver lesion in this patient is still unclear.

## 9. Patient perspective

The doctor reached a consensus on the treatment plan through sufficient communication and exchange with the patient and their family members. The patient had no adverse events during the treatment period, and the treatment effect was satisfactory. The patient’s overall survival period is now over 4 years, and the patient’s family members have accepted the treatment results.

## Acknowledgments

We thank the patient and her family for their contributions to the investigation of clinical data, serological indexes, and sample collection.

## Author contributions

**Conceptualization:** Yinhang Wu.

**Formal analysis:** Jiang Liu, Jian Liu.

**Visualization:** Jin Liu.

**Writing – original draft:** Zhiqin Chen.

**Writing – review & editing:** Jian Liu.

## Supplementary Material


