# Supplementary material for: Postoperative gastric cancer accompanied by large-cell neuroendocrine carcinoma: A case report
Source: Medicine (Baltimore). 2025 Oct 10;104(41):e44367. doi: 10.1097/MD.0000000000044367 (PMC12517824; doi:10.1097/MD.0000000000044367)

**Supplementary Materials 1**

2023-11-10 Spiral plain CT of the abdomen.


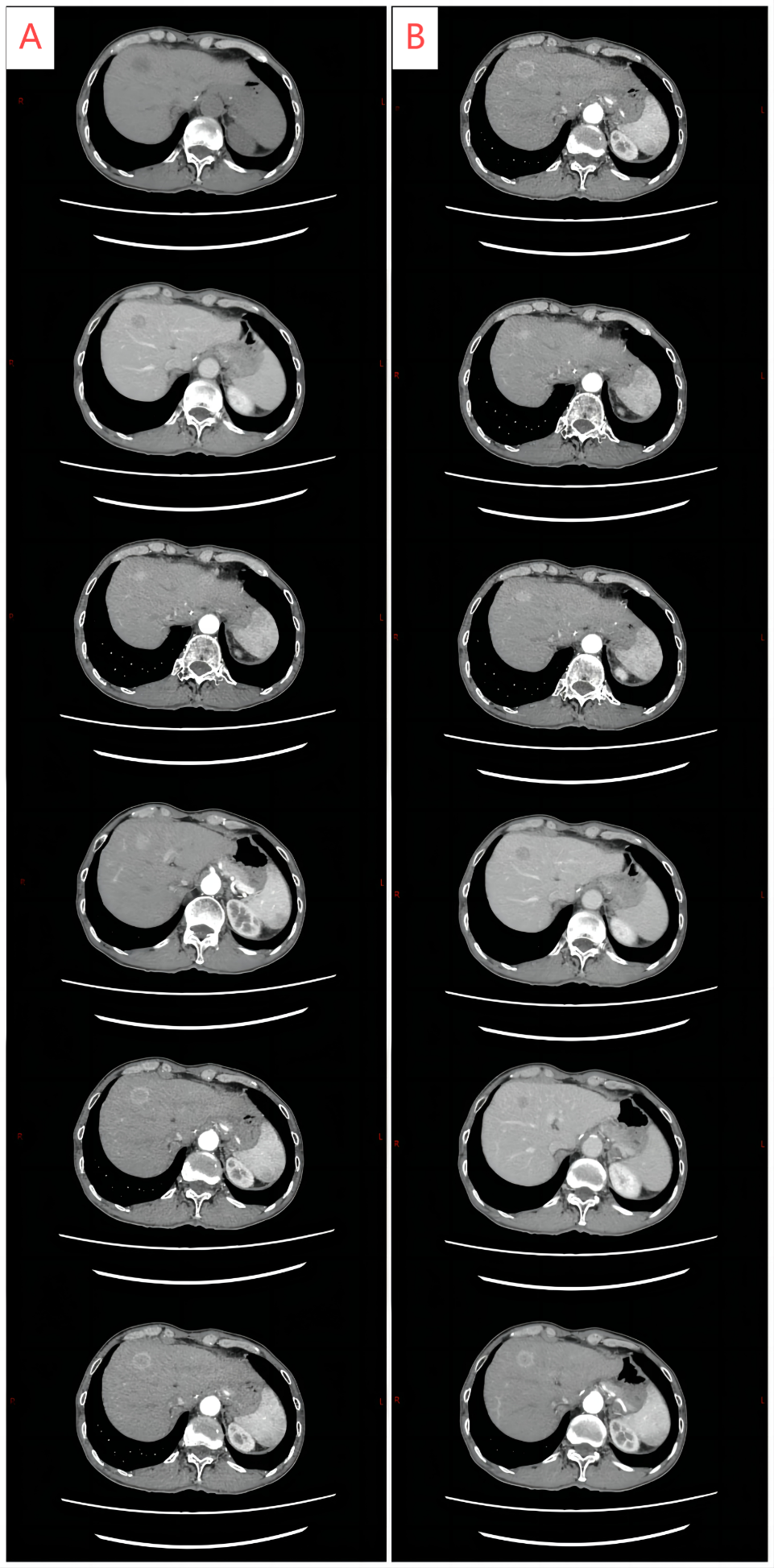


**Supplementary Materials 2**

MR images


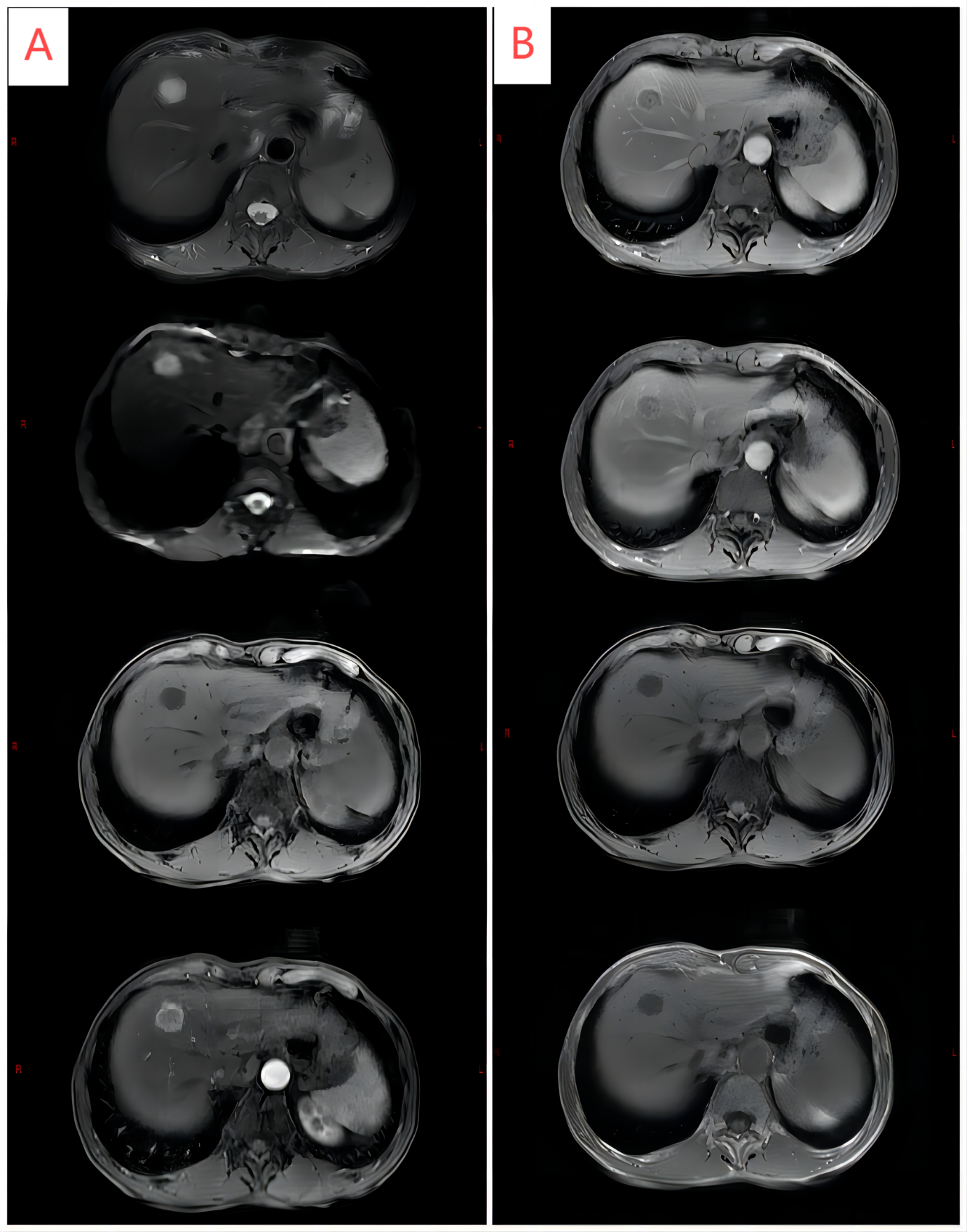


**Supplementary Materials 3**

Images of the abdomen in PET-CT images


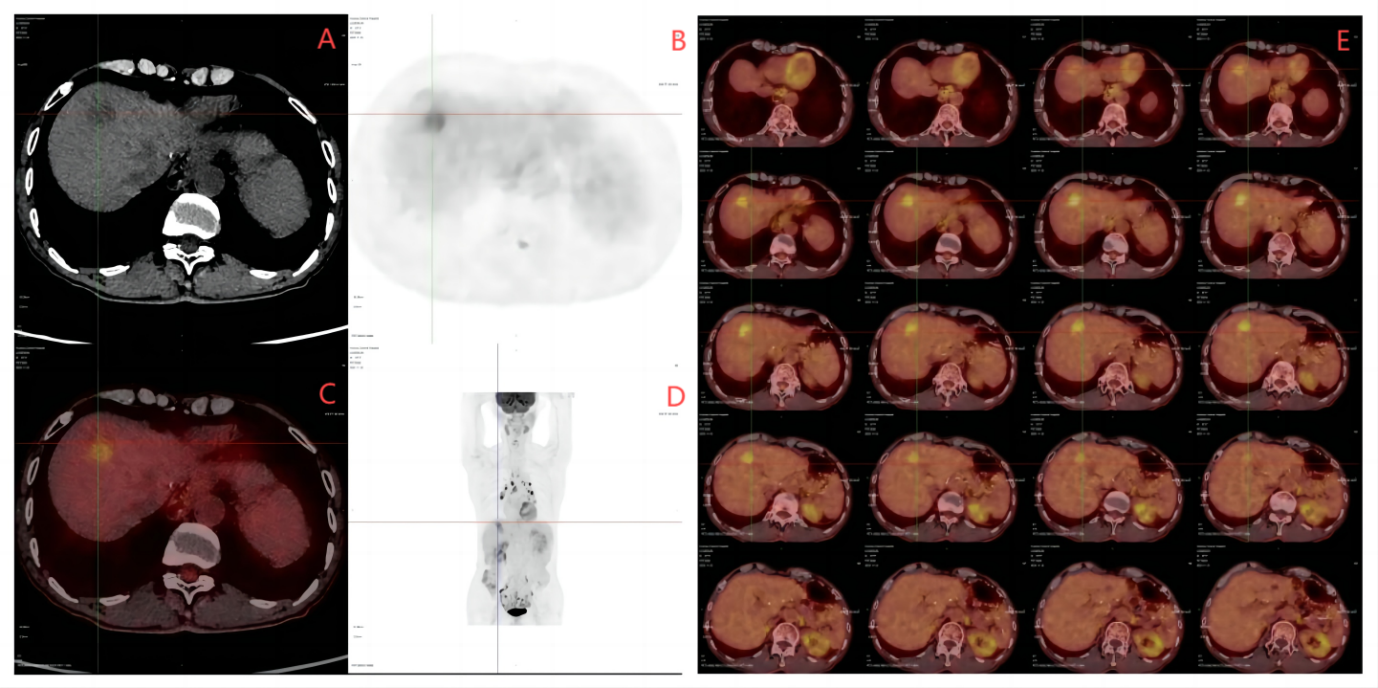

Supplement: Supplementary file 1 [file medi-104-e44367-s001.docx]
